# Computer Vision Malaria Diagnostic Systems—Progress and Prospects

**DOI:** 10.3389/fpubh.2017.00219

**Published:** 2017-08-21

**Authors:** Joseph Joel Pollak, Arnon Houri-Yafin, Seth J. Salpeter

**Affiliations:** ^1^Sight Diagnostics Ltd., Jerusalem, Israel

**Keywords:** malaria, diagnostic, computer vision, automated microscopy, fluorescent image analysis

## Abstract

Accurate malaria diagnosis is critical to prevent malaria fatalities, curb overuse of antimalarial drugs, and promote appropriate management of other causes of fever. While several diagnostic tests exist, the need for a rapid and highly accurate malaria assay remains. Microscopy and rapid diagnostic tests are the main diagnostic modalities available, yet they can demonstrate poor performance and accuracy. Automated microscopy platforms have the potential to significantly improve and standardize malaria diagnosis. Based on image recognition and machine learning algorithms, these systems maintain the benefits of light microscopy and provide improvements such as quicker scanning time, greater scanning area, and increased consistency brought by automation. While these applications have been in development for over a decade, recently several commercial platforms have emerged. In this review, we discuss the most advanced computer vision malaria diagnostic technologies and investigate several of their features which are central to field use. Additionally, we discuss the technological and policy barriers to implementing these technologies in low-resource settings world-wide.

## Background

Despite the availability of low-cost treatments, malaria caused 429,000 deaths in 2015 (1). While several diagnostic modalities exist for assessing malaria infection, a test with improved accuracy, better ease of use and speed would greatly benefit patients and clinicians. Deficiencies in the quality of test parameters (sensitivity and specificity) lead to misdiagnosis and under-treatment. Furthermore, shortcomings in the convenience and availability of testing lead to proliferation of the disease and over-treatment yielding parasite resistance. To combat the dangers of empiric treatment, several governmental health organizations now require malaria testing prior to the use of anti-malarial drugs, prompting a rise in demand to 500 million malaria tests in 2012 (2).

Quality assured human microscopy is considered the gold standard for clinical malaria diagnosis by the World Health Organization due to its ready availability. However, it is plagued by inter-user variability and inconsistency: many microscopy technicians do not assess the standard number of high-power fields, are not well-trained to recognize all forms of malaria, and the quality of manual Giemsa slide production can be highly variable (3). When compared with PCR in an asymptomatic population, human microscopy grossly underestimates the prevalence of infection (4, 5). When compared with an expert microscopist or highly accurate rapid diagnostic test (RDT), typical field microscopists can have accuracies ranging from 45 to 60% (6, 7). Moreover, in many locations there is a lack of trained microscopy experts able to conduct and implement quality assurance (8).

Automated microscopy using computer vision technologies aims to obviate the need for human microscopists by providing a consistent and accurate diagnosis without human analysis. These systems present a significant advantage over a human microscopist by potentially removing the need for training for blood film interpretation (or preparation), reducing the turnaround time, and significantly improving diagnostic performance. Furthermore, automated microscopy would maintain the benefits of microscopy over RDT by providing full species determination and parasite quantitation.

Over the last 15 years, numerous efforts have been made by both academic and industry groups to produce a fully integrated and highly accurate malaria diagnostic automated microscopy platform (9–14). A complete automated microscopy platform for malaria diagnosis is comprised of three interdependent elements: sample preparation, digital microscopy with automated scanning, and a computer vision algorithm to analyze the captured images. Previous reviews on automated microscopy for malaria have explored technical aspects relating to image analysis and acquisition. In this review, we analyze considerations involved in field application by investigating ease of sample preparation, portability, diagnostic parameters, accuracy, and limit of detection (LOD). Specifically, we discuss systems that are sufficiently developed for clinical use and have performed a trial on human malaria samples collected in the field.

## Sample Preparation

The standard protocol for malaria microscopy uses Giemsa stain to identify parasites in peripheral blood films. While Giemsa staining produces a very clear malaria stain, the protocol is arduous, requiring smearing the blood correctly, a short immersion in methanol, followed by a longer immersion (usually 10–45 min) in Giemsa solution. The slide is then washed and dried, often requiring an hour or more to complete. Factors such as the pH of the water used and quality of Giemsa stain are critical (15).

Many automated microscopy systems have built their platforms around Giemsa staining (16–18). While this approach adapts to the current laboratory standard, it poses significant challenges for the algorithm construction as technicians use different staining protocols and reagents, yielding disparate images. One system, studied by Delahunt et al. (19), used Giemsa slides prepared at seven different locations to create a varied library of images to train the machine-learning based algorithm, aiming to reduce the impact of variable stain quality. This approach enables the retention of current low-cost staining techniques, but poses greater challenges in algorithm development and its accuracy will remain somewhat dependent on the quality of the technician-prepared blood film.

In contrast, several systems have used fluorescent stains to yield greater diagnostic accuracy due to lower stain variability and higher contrast intensity. The use of a fluorescent dye also provides for a quicker sample preparation process, as live fluorescent stains can occur in less than 1 min (versus the much longer process described above). The Cellscope, developed by Partek (20), uses a UV-excited fluorescent stain. Alternatively Vink et al. used Acridine Orange, a quick acting, and already proven fluorescent stain (21). Indeed, Guy et al. (22) provide an extensive assessment of fluorescent dye staining of *Plasmodium falciparum* by assessing 22 fluorescent nucleic acid specific dyes, as well as their capability for co-stain with Giemsa. They determined Syber Green 1 to be the superior overall fluorescent stain in terms of both co-staining and intensity. The SYTO family of dyes also excelled in its capacity to stain cultured malaria positive red blood cells (RBCs). Additional fluorescent-based systems have used a dye combination to achieve accurate staining such as the *Parasight* system which makes use of multiple dyes in the 370 and 475 nm excitation imaging ranges (23–25).

Notably when using fluorescent staining, custom design slide carriers were also required for each of these units to accommodate the live intra-vital staining process. Carrier types include both one-step microfluidic cartridges that internally stain whole blood samples, as well multi-step units where the blood mixed with a diluent, transferred *via* pipette, and then automatically dispersed. While microfluidic cartridges provide an advantage by potentially creating a one-step staining process, they significantly increase design and manufacturing cost.

## Portability

When assessing the viability of the automated microscopy malaria diagnostic systems, portability remains a significant factor. Diagnosis needs to be accessible near the patient, ideally at the point of care, as rapid identification, and treatment is critical to successful disease management (26, 27). Such access has been made feasible in recent years for remote populations where malaria is often prevalent, through the advent of RDTs. Indeed, RDTs are extremely useful in remote locations as they do not require electricity, are handheld, and require minimal training. Microscopy requires lab space for staining, a large electricity-powered microscope, and trained personnel. However, it remains a staple of diagnosis in large clinic settings, where the throughput of patients is high and species and parasite burden quantification matter. The use of portable battery-powered digital microscopes would obviate the need for technicians and some lab space, but simplified sample preparation such as self-staining slides are required to take to more remote areas at scale.

Potential power sources for an automated microscopy system include battery packs or solar energy. The Cyscope, which is an upright microscope, uses a rechargeable battery pack. Currently, there are no automated malaria microscopes with a portable power source. However, precedence for such devices exists; several CD4+ T cell devices used in the treatment of HIV such as the BD FACSPresto and Alere Pima can be powered by battery packs that last for approximately 6 h.

To reduce the power demands and add mobility to automated microscopes, several studies have focused on portable malaria diagnostic microscopes using mobile phone systems. An early system used a 20× wide field microscope with a white LED light source to illuminate brightfield images on Giemsa stain slides (28). Recent studies have updated the methodology by using polarized white light microscopy. Pirnstill and Coté have shown the ability to visualize Giemsa stained blood smears at a 40× objective with an iPhone (29). A second, already commercialized, system for smartphone microscopy is the X-rapid system, which is an LED and 10× lens attachment to a typical smart phone (30). The volume scanned is set by the user by the number of high power fields, RBCs, or WBCs. Additional data will be required to assess whether these systems could replace RDTs or high quality microscopy in the future.

## Device Limit of Detection

Studies have shown that the LOD for microscopy and RDTs range between 100 and 500 parasites/μL of blood depending on the technician and brand of RDT (31, 32). On the other end of the spectrum, PCR assays have been reported to have LOD’s in the range of 1–5 parasites/μL (33, 34). Improving the LOD for malaria diagnostic assays has been set as a primary goal by the WHO for next generation of malaria diagnostic systems. In a recent evidence review group, it was suggested that a system able to detect infections of 2 parasites/μL is highly desirable to identify asymptomatic patients (35). Furthermore, in the context of population screening in areas with low prevalence, evaluations have shown that test with an LOD of 1–2 parasites/μL could potentially double detection rates compared with RDTs or microscopy (4, 5). Recent studies have confirmed the need for detecting very low levels of infection, showing that that in certain populations between 20 and 50% all infections could be sub-microscopic (36, 37).

An important value proposition of computer vision systems is the potential to significantly lower the LOD. The LOD is a function of the total volume of blood scanned, since in malaria cases with low parasitemia, infected RBCs are rare. While a standard microscopist aims to scan approximately 10,000 erythrocytes at high resolution on a thin smear, automated microscopes have the potential to scan an entire blood film (~2 μL which is ~10,000,00 RBCs) with speed determined by image magnification, acquisition, and processing speeds. One of the earliest computer vision automated microscopists, the World Health Technology autoanalyzer (16) reported on a prototype system with an estimated limited of detection of 140 parasites/μL. These results were produced using a platform that scanned with a 40× objective and recorded 800 high powered fields in 5 min. Recently, a Global Good Fund prototype showed an LOD in the range of 100 parasites/μL for *P. falciparum* while scanning 0.2 μL of blood.

While these two platforms use giemsa smear preparations, platforms based on fluorescent stains are also able to provide low LODs. A platform designed by Vink et al. using a live intra-vital cartridge stain is capable of analyzing 0.47 μL of blood, potentially analyzing 2.5 million RBCs, and yielding a projected LOD of 10 parasites/μL within 15 min (21). A second fluorescent system, the *Parasight* malaria detection device, showed an LOD of under 100 parasites/μL (25). Limits of detection on these devices should reduce with further algorithm development, though the LOD will eventually be limited by the noise floor caused by artifacts from film preparation and staining.

## Device Parameters and Performance

Standard microscopy for malaria diagnosis provides species differentiation, parasite density, and identification of gametocytes, at a range of accuracy dependent on the quality of the film, and the microscope and the proficiency of the microscopist (38–41). All three of these pieces of data are helpful to make accurate clinical treatment decisions (42, 43). Specifically, different species are treated with different anti-malarial drugs, while high parasite densities can indicate the need for emergency treatment and hospitalization, and the presence of gametocytes is important when looking to understand drug effects and transmission patterns. Besides microscopy, all other modalities cannot provide all of these parameters. RDTs provide diagnosis through detection of parasite-derived antigens with overall performance generally better for *P. falciparum* than *P. vivax* (44, 45). *P. ovale* or *P. malariae* are moderately differentiated by some RDTs. No RDTs can provide parasite density or identification of gametocytes. PCR-based technologies provide highly accurate diagnosis and species differentiation, and can be quantitative, but cannot identify gametocytes.

A computer vision system should be able to offer these three features. However, given the rarity of some malaria type cells, assembling the proper database to train the algorithm is challenging. For example, while *P. falciparum* and *P. vivax* are found in numerous locations throughout the world, *P. ovale* comprises approximately 5% of all cases (46), and *P. malariae* is even scarcer (47). Extensive and targeted data collections are therefore required to construct a computer vision platform that can provide classification for all types of malaria. An additional feature that automated microscopy and human microscopy may offer that these other options lack is the ability to catch basic hematologic abnormalities such as severe sickling, high numbers of blast cells, or an abnormal white cell differential.

Where routine Giemsa-stained slides are used, variations in staining (and in film thickness) can add further challenges which must be dealt with in distinguishing parasite and hematological features. This requires large libraries of films to be developed incorporating the full cross-section of such variation. This is dealt with in some systems using alternative staining techniques (fluorescence) or by the use of self-spreading and self-staining slides, but this has not been achieved with Romanowski-type stains (of which Giemsa is the most common). The problem of slide quality is a handicap of systems aimed at reading routine malaria blood films using classical smears, and the advantages of low-sample preparation cost and reduced workflow change are weighed against the laboratory skills and capacity required.

## Discussion

Computer vision malaria diagnostic systems have moved from early stage research applications to mature commercial platforms that are now available in diagnostic laboratories. Initial novel microscopy applications using fluorescent dyes that did not provide a final diagnosis (such as QBC and Cyscope) succeeded in reaching the market, yet did not achieve wide acceptance, potentially since the human microscopist was still needed for verification. Moreover, they are not stand-alone systems and still require several pieces of ancillary equipment for operation. The automated microscopy computer vision systems discussed in this review (see Table 1 for summary), remove the human microscopist from the process and sometimes offer greatly simplified sample preparation.

**Table 1:** Summary of advance computer vision systems discussed in this review.

	WHT	Automatic vision-based system	Autoscope	*Parasight*	Automated diagnostic app
Developer	Hydas World Health	Philips Group	Global Good	Sight Diagnostics	X-Rapid
Portable	No	No	No	No	Yes
Stain type	Giemsa	Fluorescent	Giemsa	Fluorescent	Giemsa
Automated scanning	No	Yes	Yes	Yes	No
Scanning time	5 min	15 min	20 min	4 min	Depends
Commercially available	No	No	No	Yes	Yes
Publication	Prescott et al. (16)	Vink et al. (21)	Delahunt et al. (19)	Eshel et al. (25)	NA

The systems mentioned here are designed around either existing (giemsa smear) or novel sample preparation setups (cartridge and fluorescent stain) which produce differing target performance parameters. The stated consensus among all the systems is to achieve an LOD under 150 parasites/μL. This LOD would parallel the performance of the above average field microscopist and would offer the advantage of having a consistent automated technology that is available without issues of fatigue or retraining. Achieving detection at <5 p/μL remains challenging due to the small amount of blood analyzed by all of these platforms (<1 μL) and the presence of parasite like objects in the blood including Howell–Jolly bodies, platelets on RBCs, and staining debris. Critical to confirming the performance of these systems will be large scale multi-site studies to validate the technologies in a range of epidemiological settings.

A commercial computer vision malaria diagnostic device would also benefit from additional assays to create a multiplexed platform. Computer vision systems for Tuberculosis have previously been developed (48–50). The TBDx system by applied visual sciences uses proprietary algorithms to detect and count acid fast bacilli at 40× magnification. Digital cytology systems have been extensively used in screening for cervical cancer (51, 52). Combining these particular tests into a unified platform would provide a particularly attractive device in developing world settings where these diseases are highly prevalent and poorly diagnosed.

Widespread adoption of digital microscopy and computer-aided will require adequate demonstration of excellent performance, regulatory acceptability, and significant ease of use. Some devices, such as *Parasight* and X-Rapid have already found a commercial market. Automated microscopy has the additional advantage of often providing images to clinicians, disambiguating what might otherwise feel like a black box diagnostic technique. With the case of malaria, the use of manual microscopy has retained a place despite frequent poor performance partly due to habit and tradition, but largely because it offers transparency and clinically relevant information that other techniques lack. If emerging digital systems can overcome the failings of manual systems while retaining their benefits, they should be able to grow the microscopy market.

## Author Contributions

JP, AH-Y, and SS all composed the manuscript.

## Conflict of Interest Statement

JP, AH-Y, and SS are employees of Sight Diagnostics and declare a conflict of interest.
